# A novel ventricular septal reconstruction device for post-myocardial infarction ventricular septal rupture: preclinical and first-in-human study results

**DOI:** 10.3389/fcvm.2026.1892390

**Published:** 2026-09-09

**Authors:** Changjing Huang, Nan Cai, Youqian Li, Yao Yao, Zexun Zhu, Weike Wu, Kaibing Ren, Siwei Wu, Jingfeng Liu, Jingyuan Hou, Haifeng Hong, Hanlin Li, Zhihui Hu, Zhixiong Zhong, Wei Zhong

**Affiliations:** 1 Fifth Department of Cardiovascular Medicine, Meizhou People’s Hospital, Meizhou, Guangdong, China; 2 MicroPort® CardioFlow Medtech Corporation, Shanghai, China; 3 MicroPort® CardioAdvent Medtech Corporation, Shanghai, China; 4 Department of Ultrasound, Meizhou People’s Hospital, Meizhou, Guangdong, China; 5 Guangdong Engineering Technological Research Center of Molecular Diagnosis in Cardiovascular Diseases, Meizhou, China; 6 Institute of Cardiovascular Disease, Meizhou Clinical Institute of Shantou University Medical College, Meizhou, Guangdong, China; 7 Meizhou Key Laboratory of Cardiovascular Translational Medicine, Meizhou, China

**Keywords:** first-in-human study, post-myocardial infarction ventricular septal rupture, preclinical study, structural heart intervention, transcatheter closure, ventricular septal reconstruction device

## Abstract

**Background:**

Post-myocardial infarction ventricular septal rupture (PIVSR) is a severe mechanical complication of acute myocardial infarction (AMI) with a poor prognosis. Current therapeutic strategies—including pharmacotherapy, surgery, and off-label use of congenital occluders—are associated with inherent limitations.

**Objectives:**

The primary objective of this study was to evaluate the procedural feasibility, preliminary safety, and short-term performance of a novel dedicated Ventricular Septal Reconstruction Device (VSRD) for the treatment of PIVSR.

**Methods:**

This study employed preclinical animal studies and a prospective first-in-human (FIM) clinical study. For the animal experiments, VSD-like defect creation was attempted in 14 Labrador retrievers; 11 underwent device-placement attempts, and 10 completed implantation and detachment with the VSRS 3226 device. Five animals were assessed at day 1 and five at day 60. The canine model was used to evaluate device deliverability, deployment, anchoring, shunt reduction, and short-term local tissue response in a VSD-like defect model rather than to reproduce infarct-related PIVSR pathology. For the first-in-human study, 4 PIVSR patients were enrolled; the transjugular approach was adopted, guidance was provided by transthoracic echocardiography (TTE) combined with left ventricular angiography, and follow-up duration exceeded 6 months.

**Results:**

In animal experiments, 11 of 14 animals proceeded to device-placement attempts, and 10 completed implantation and detachment; no device structural malfunction or delivery-system failure was recorded. Minor residual leakage was qualitatively observed in 4/5 animals at day 1, whereas no leakage was detected in the separate five-animal day-60 group; tissue coverage consistent with endothelialization was observed at day 60, and no adverse events occurred among implanted animals during follow-up. In the first-in-human study, implantation was completed in all four patients; residual shunts were ≤2 mm during follow-up, and no high-grade atrioventricular block or permanent pacemaker implantation occurred.

**Conclusions:**

The VSRD demonstrated procedural feasibility and acceptable short-term safety and performance in this preclinical and first-in-human experience, in selected patients and a VSD-like animal model. Larger-scale clinical studies with longer follow-up are needed to evaluate its long-term safety, effectiveness, durability, and comparative performance. These findings should be interpreted cautiously because the clinical cohort was small, highly selected, and non-comparative.

## 1 Introduction

Post-myocardial infarction ventricular septal rupture (PIVSR) is one of the most severe mechanical complications of acute myocardial infarction (AMI), with a global incidence of 0.17% to 0.44% and an extremely poor prognosis—patients treated with only pharmacological therapy have a 1-month survival rate of merely 6% and a 1-year survival rate of less than 3%; as the global burden of cardiovascular diseases continues to increase and the population ages, the number of PIVSR patients is on the rise (1, 2). Current treatments for PIVSR include pharmacological therapy, surgical intervention, and transcatheter closure: pharmacological therapy only serves as basic supportive care and is associated with a poor prognosis; surgical intervention, while effective, is highly invasive and has a high mortality rate, with the incidence of significant residual shunts after surgery ranging from 10% to 20% (1, 3). Transcatheter closure currently relies mainly on occluders designed for congenital ventricular septal defects. Because PIVSR is characterized by friable septal tissue and often irregular or multiple defects, the anatomical adaptability of these devices may be limited. In selected patients with high surgical risk, suitable anatomy, or failed surgical repair, transcatheter closure may be considered as an alternative to surgery (4–6). A recent meta-analysis comparing surgical and interventional treatments showed no significant difference in long-term mortality between the two approaches (7). The Abbott Amplatzer P.I.VSD has obtained humanitarian device exemption (HDE) approval from the FDA, and its clinical studies have demonstrated a technical success rate of 90.5% and a 1-month postoperative survival rate of 47.6% in enrolled patients and high-risk patients (8). Despite these advances, effective closure of PIVSR remains an unmet clinical need, particularly in patients with large or complex defects who are unsuitable for surgery or currently approved devices (1, 2, 8). The “Ventricular Septal Reconstruction Device,” an investigational medical device developed by MicroPort Scientific Corporation (MicroPort Medical), is specifically designed for PIVSR, and this paper reports the short-term results of this device in animal models and first-in-human studies. The present investigational device was developed with a small-waist large-disc configuration, polyethylene terephthalate (PET)-covered discs, hollow guidewire-compatible delivery, and a transjugular delivery pathway, which distinguish it from conventional off-label congenital VSD occluders mainly at the device-design and procedural levels.

## 2 Materials and methods

### 2.1 Device design

**Figure 1:**
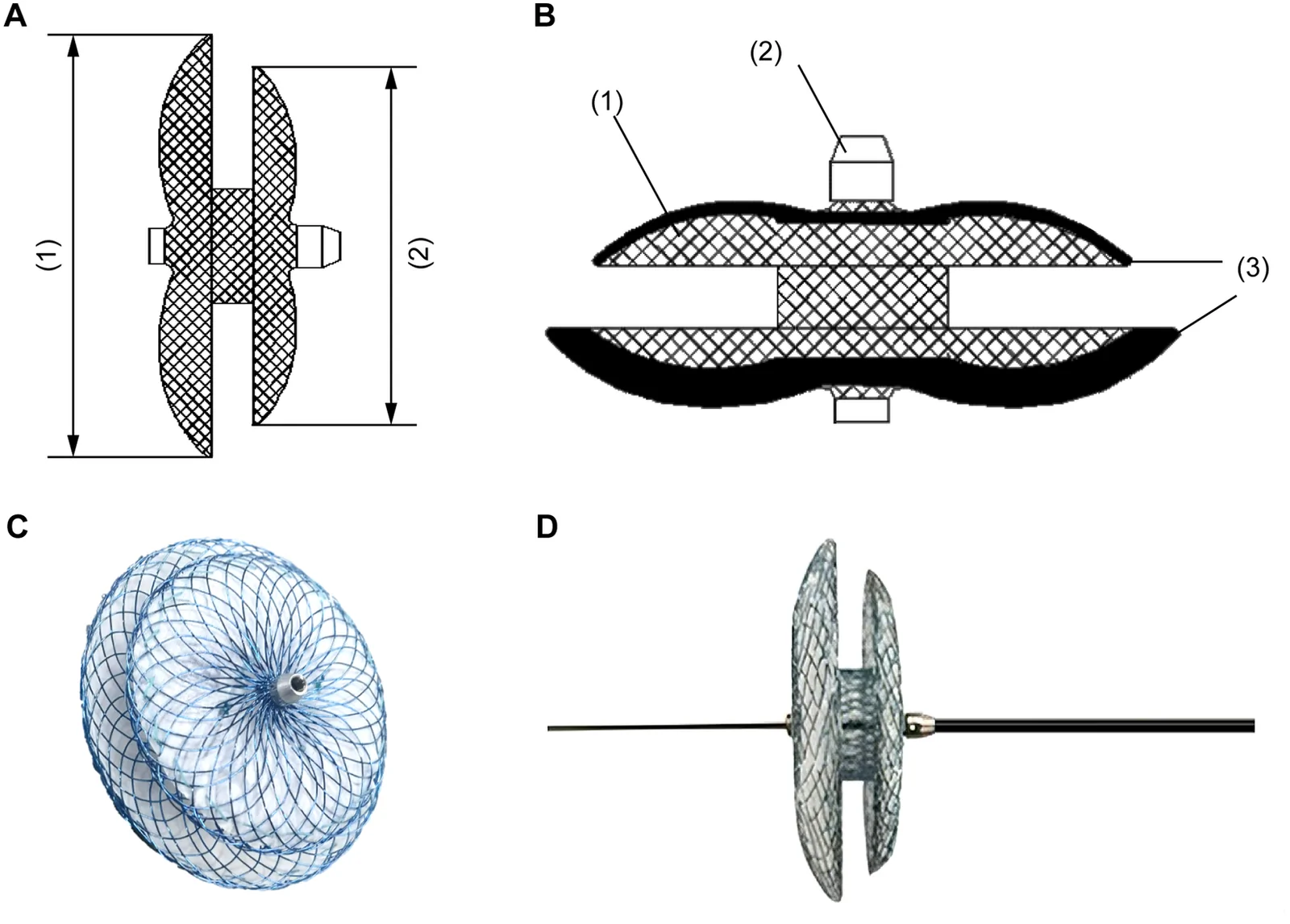
Schematic diagrams and actual images of the VSRD**. (A)** Schematic cross-sectional view of the VSRD: (1) Left disc, (2) Right disc. **(B)** Same cross-sectional view (showing): (1) Metal mesh frame, (2) Proximal threaded head, (3) Covering membrane and sutures. **(C)** Device appearance schematic. **(D)** Actual device image: Demonstrating the hollow design that allows passage of a guidewire. Images provided by MicroPort (https://microport.com/).

The VSRD is specifically designed for PIVSR, and its main structure comprises a reconstruction device main body and a matching delivery system. The main body consists of a “double-disc single-waist” framework woven from medical nitinol wires; the inner sides of the left and right discs are each fixed with a layer of PET membrane via sutures to block shunts, and a stainless steel bolt head is arranged at the center of the right disc to connect with the delivery rod. The delivery system includes a delivery rod, a guide, a set of delivery sheaths, and a loader, which are used for device delivery, positioning, vascular access preprocessing, and device loading, respectively. The overall design of the VSRD takes adapting to the pathological characteristics of PIVSR as the core principle, adopting a “small-waist large-disc” structure combined with a double-disc clamping and fixation mechanism — this differs from the design logic of traditional ventricular septal occluders that rely on radial support force from the waist. Its waist is designed to be thin, with the aim of reducing the contact area and radial loading on the fragile myocardium around the rupture; together with the large-diameter PET-covered discs, this configuration was intended to increase the sealing surface on the surrounding septal tissue, with the aim of reducing compressive damage to the fragile myocardium in the infarcted area. Meanwhile, the device adopts a hollow guidewire-compatible design, supporting the full-course guidewire retention operation and reducing the risk of tissue damage caused by intraoperative device replacement and secondary track establishment. In addition, the surgical approach has been optimized, with interventional procedures performed via the jugular vein approach to further reduce the risk of vascular access-related damage, and the overall design was developed to address the clinical needs of PIVSR patients (who are in critical condition with fragile myocardial tissue); the salient characteristics of the VSRD design are shown in Figure 1. The device is available in three sizes: VSRS 3226, VSRS 3628, and VSRS 4032, with the left disc diameters of 32 mm, 36 mm, and 40 mm, respectively, and the corresponding right disc diameters of 26 mm, 28 mm, and 32 mm, so as to meet the needs of different perforation sizes. The delivery system used with the device is a universal 14F system, and device selection follows the predefined rule of left-disc diamete*r* = 2 × maximal defect diameter+8 mm, corresponding to VSRS 3226 for defects ≤12 mm, VSRS 3628 for 12–14 mm, and VSRS 4032 for 14–16 mm. Before detachment, device configuration, disc apposition, stability, and residual shunt are assessed by echocardiography and left ventricular angiography. If residual shunt or device adaptation is considered unsatisfactory, the device can be partially or completely recaptured, redeployed, or exchanged before release.

### 2.2 Animal experiment

#### 2.2.1 Animal model

From December 2022 to March 2023, 14 adult Labrador retrievers underwent attempted creation of VSD-like septal defects; three animals did not proceed to device implantation because the puncture/defect-creation positions were excessively high and repeat defect creation was not feasible; in one additional animal, device placement was terminated because the device position was excessively high, was judged to interfere with the mitral and tricuspid valve leaflets, and mitral regurgitation was observed; 10 animals with anatomically suitable VSD-like defects were eventually included in the final analysis (8 males and 2 females). The mean preoperative body weight of the experimental dogs was 31.45 ± 2.57 kg (range: 28–35 kg). One day before the procedure, the dogs received oral aspirin (100 mg) and clopidogrel (75 mg); intraoperatively, 1 g of ceftriaxone sodium was administered via intravenous bolus. For the group with 60-day postoperative follow-up, 0.5 g of amoxicillin was given orally once daily for 3 consecutive days, and continuous oral administration of aspirin and clopidogrel was maintained until the end of the experiment; the group with 1-day postoperative follow-up received no additional medication, as these dogs were euthanized on the day after surgery. All animals received humane care, and the experimental procedures were conducted in compliance with the 2011 Guide for the Care and Use of Laboratory Animals. The study was approved by the Institutional Animal Care and Use Committee (IACUC) of Meizhou People's Hospital with approval number 2022-061.

#### 2.2.2 Animal procedure

This procedure was performed under general anesthesia. Anesthesia was induced via intramuscular injection of Zoletil 50 (100 mg, approximately 2.9–3.6 mg/kg based on the preoperative body-weight range), followed by inhaled isoflurane (2%, adjusted as needed within the protocol range of 1%–5%; oxygen flow, 1 L/min) to maintain the anesthetic state. After successful anesthesia induction, orotracheal intubation under direct vision was performed to connect to a ventilator; during the procedure, real-time monitoring of heart rhythm, blood pressure, and oxygen saturation was conducted, with monitoring data recorded every 15 min, and 1 g of ceftriaxone sodium was administered intraoperatively for infection prophylaxis. The jugular vein was used as the route for ventricular septal puncture and device delivery, while the left femoral artery was punctured to serve as the route for guidewire capture and track establishment.

**Figure 2:**
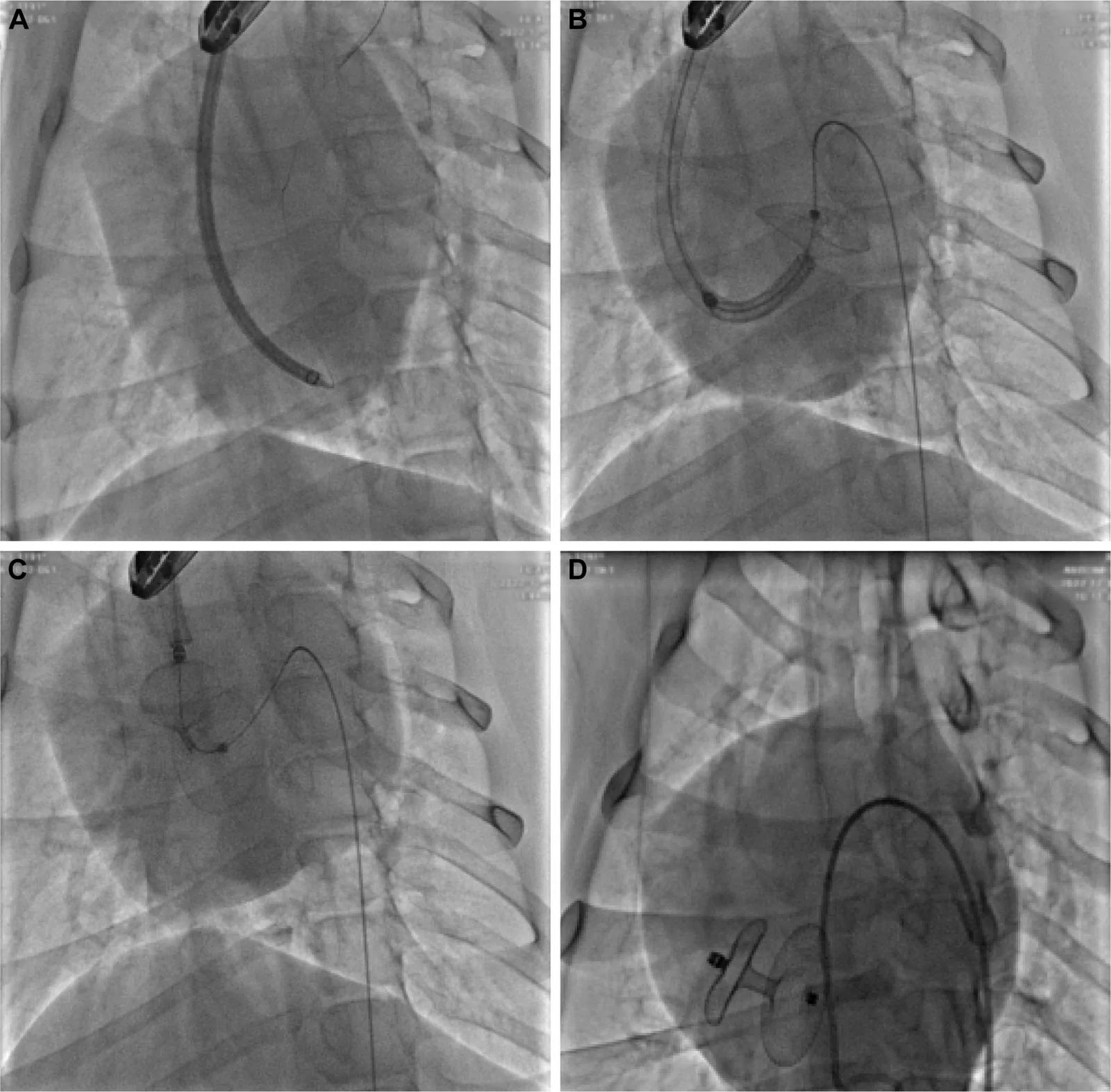
Fluoroscopy-Guided ventricular septal defect (VSD) creation and subsequent closure with the VSRD in an animal model. **(A)** Under the support of a ventricular septal puncture sheath, the trailing end of a guidewire was successfully passed through the ventricular septum and accessed the left ventricle. **(B)** The left disc was deployed, and the delivery sheath was withdrawn until the left disc apposed the ventricular septum. **(C)** The right disc was deployed. **(D)** The delivery system was separated from the occluder, completing device deployment.

Under the guidance of a cardiovascular ultrasound system (manufactured by Philips Ultrasound B.V.) and a digital subtraction angiography (DSA) machine (manufactured by Beijing GE Huadun Medical Co., Ltd.), ventricular septal defect (VSD) creation was performed, and the puncture thickness was measured and confirmed via ultrasound or angiography. After successful puncture, a 14F delivery system was advanced for secondary dilation of the puncture site; following the removal of the dilator, a balloon was advanced along the guidewire, and the balloon was inflated for tertiary dilation, after which the balloon was removed upon completion of defect creation. The tracking guidewire was retained after defect creation, and the VSRD was rinsed and assembled. Due to the smaller size of the experimental dogs’ hearts compared to that of humans, the VSRS 3226 specification device was selected for all dogs. The device was advanced along the guidewire from the jugular vein into the left ventricle; first, the left disc was deployed to appose the ventricular septum on the left ventricular side, then the left disc was gently pulled with the delivery rod and the right disc was deployed in a retracting manner, allowing the left and right discs to clamp the ventricular septum. Subsequently, ultrasound was used to confirm the device position, a pigtail catheter was advanced for left ventricular angiography, and after DSA confirmed no significant leakage, the delivery rod was rotated counterclockwise to disconnect and complete device detachment. Figure 2 depicts the VSD occlusion procedure with the VSRD in an animal model. In this preclinical experiment, the septal defect was intentionally generated by transjugular puncture followed by stepwise dilation under DSA and echocardiographic guidance. The created defects were not designed to reproduce myocardial infarction, necrosis, and remodeling; rather, they were used to evaluate device deliverability, deployment, anchoring, shunt reduction, and short-term tissue response in a reproducible VSD-like environment. Defect size and position were assessed for compatibility with the selected device, but the defects were not sculpted to mirror the final device configuration. Accordingly, the animal findings should be interpreted as preclinical feasibility and short-term biocompatibility data in a VSD-like model, not as direct evidence of performance in infarcted and friable myocardium.

#### 2.2.3 Evaluation protocol

Device success was defined as follows: the VSRD was implanted into the target position along the track, with the left and right discs apposing the two sides of the ventricular septum respectively; DSA confirmed no significant leakage, ultrasound verified no positional deviation, the device could be successfully detached from the delivery rod, and the device structure remained intact.

The scope of adverse events included device migration, dislodgement, or structural damage; thrombosis and vegetation formation on the device surface; cardiac valve regurgitation and ventricular septal tissue damage; abnormal lesions in other organs such as the liver and spleen; and postoperative reactions including hemolysis, wound infection, and abnormalities in mental state and appetite.

Immediately after device implantation, DSA and ultrasound were performed on all 10 experimental dogs to evaluate device position and shunt. Five of the experimental dogs were euthanized on postoperative day 1, with complete blood count (CBC), blood biochemistry, and endpoint imaging conducted before euthanasia; the other 5 experimental dogs underwent endpoint imaging, CBC, and blood biochemistry on postoperative day 60, followed by euthanasia. At each predetermined endpoint, euthanasia was performed under anesthesia by intravenous administration of 10 mL of 3 mol/L saturated potassium chloride (KCl) solution. The evaluation indicators included device success rate, residual-shunt status assessed by DSA and echocardiography, adverse event rate, thrombosis rate, and endothelialization rate. After euthanasia, gross observation of the heart and major organs was performed via dissection; for the 60-day group, additional histopathological section examination of the tissue surrounding the device was conducted.

### 2.3 First-in-human study

#### 2.3.1 Study design

This was a prospective, single-center, exploratory clinical study that enrolled 4 patients with PIVSR at Meizhou People's Hospital in 2024. The hospital has extensive experience in transcatheter cardiovascular intervention, holds qualifications for performing percutaneous coronary intervention (PCI), and is capable of conducting core examinations such as cardiac ultrasound and left ventricular angiography. Additionally, it can provide mechanical circulatory support including intra-aortic balloon pump (IABP) and extracorporeal membrane oxygenation (ECMO) to meet the treatment needs of critically ill patients.

This study was approved by the Ethics Committee of Meizhou People's Hospital (Approval No.: 2023-A-71), and written informed consent was obtained from all subjects. The VSRD was used in the study, and transjugular PIVSR closure was performed under the guidance of TTE and left ventricular angiography. Color Doppler ultrasound was used to visualize the shunt and measure the shunt diameter, where the defect diameter was defined as the maximum measured diameter of the shunt. Cardiac ultrasound examinations were performed on the patients immediately after surgery, before discharge, at 30 days, 3 months, and 6 months postoperatively. All patients received adequate pharmacological treatment; the specific regimen was formulated by the researchers based on the patients’ actual conditions, with a treatment course of 6 months. In addition, source CRFs recorded the underlying coronary artery disease history, culprit vessel, prior coronary intervention, cardiac function class, and infection/thrombosis-relevant baseline laboratory and clinical variables. Antiplatelet/anticoagulation use and infection/endocarditis prophylaxis were determined according to institutional clinical standards and patient condition.

Clinical trial registration: This medical device clinical trial was filed with the provincial medical products administration in China (filing No. Shanghai Medical Device Clinical Trial Filing 20240043; filing date: March 11, 2024). The filed study was titled FIRST-RESCUE FIM: preliminary evaluation of the feasibility of transcatheter VSRD intervention for ventricular septal perforation after acute myocardial infarction, sponsored by MicroPort Investment Holding Co., Ltd. and conducted at Meizhou People's Hospital; the planned project period was February 6, 2024 to December 31, 2026.

Adverse events defined in this study included death, air embolism, cardiac arrest, arrhythmia, device migration or dislodgement, infection, vascular access complications, and valvular insufficiency.

The primary procedural endpoint was intraoperative immediate device success, defined as successful device implantation with proper positioning and successful withdrawal of the sheath, without residual shunt requiring emergency surgical intervention. This criterion was intended to identify residual shunting requiring surgical rescue; residual shunt was otherwise assessed separately by TTE. Safety endpoints included death, adverse events, and device-related complications during follow-up. Additional clinical and imaging assessments included procedural time, cardiac function assessment, and residual shunt on cardiac ultrasound. According to the prespecified clinical protocol, complete ventricular septal reconstruction at 30 days was defined as healing of the original defect with a residual shunt ≤2 mm on echocardiography. According to the clinical protocol, echocardiographic results relevant to efficacy assessment were evaluated by two independent cardiac imaging physicians.

#### 2.3.2 Patients

Inclusion criteria for patients were as follows: age ≥18 years and <75 years; diagnosis of PIVSR; maximum diameter of ventricular septal rupture ≤16 mm; presence of left-to-right shunt at the ventricular level, with confirmed discontinuity of the ventricular septum via imaging examinations such as ultrasound, left ventricular angiography, or computed tomography (CT), and concurrent presence of a holosystolic murmur audible at the 3rd to 4th intercostal space on the left sternal border during physical examination; stable condition after 2 weeks of support with intensive pharmacological treatment, IABP assistance, and/or ECMO; voluntary participation in the study, with patients and their family members requesting transcatheter ventricular septal closure treatment, signing the informed consent form, and being willing to undergo long-term follow-up. Clinical stabilization after intensive support was a prespecified eligibility criterion for this first-in-human feasibility study, intended to define an initial population in whom transcatheter intervention could be performed under controlled conditions. Key anatomical exclusions included perimembranous defects or defects close to the aortic/mitral valve or chordae, poor defect rims, particularly absence of an anterior or posterior rim, multiple perforations unsuitable for interventional reconstruction, free-wall rupture or chordal rupture, and any intracardiac/access-route thrombus or active condition making percutaneous intervention inappropriate.

#### 2.3.3 Procedure

The procedure was performed under local anesthesia. During the procedure, unfractionated heparin (100 IU/kg) was administered intravenously to maintain an activated clotting time (ACT) > 250 s; concurrent left ventricular angiography and TTE were conducted intraoperatively—left ventricular angiography was used to identify the location of the ventricular septal perforation, color Doppler ultrasound was employed to clearly visualize the abnormal shunt flow at the perforation site, and to accurately measure the maximum diameter of the shunt flow (this diameter was defined as the perforation diameter).

The size of the device used for occlusion was determined based on the perforation diameter: the diameter of the device's left disc was calculated as twice the perforation diameter plus 8 mm. Specifically, if the perforation diameter was ≤12 mm, a device with a 32-mm left disc was selected; when the perforation diameter ranged from 12 to 14 mm, a device with a 36-mm left disc was used; and for perforation diameters between 14 and 16 mm, a device with a 40-mm left disc was chosen. The device was implanted via the right jugular vein; during the procedure, a venous-arterial guidewire track was first established. After assembling the device with the delivery system, the device was steadily delivered to the perforation site by the delivery sheath under the guidance of a dilator and guidewire. Its deployment process relied on the clamping effect of the double-disc structure to securely fix the device to the ventricular septum, and the entire operational procedure was standardly completed under close imaging monitoring to ensure the device was placed in the correct position and closely adapted to the tissues surrounding the perforation.

### 2.4 Statistical analysis

Because of the exploratory nature of this study and the small first-in-human sample size, all clinical data were summarized descriptively. Continuous variables were presented as individual values and, where appropriate, as ranges; categorical variables were presented as counts. No formal normality testing or between-group statistical comparison was performed.

## 3 Results

### 3.1 Results of animal experiments

#### 3.1.1 Outcomes of VSD closure

Among the 14 animals that underwent attempted VSD-like defect creation, 10 had anatomically suitable created defects and completed device implantation and detachment for final analysis. The remaining four animals were not included in the final analysis: three did not proceed to device implantation because the defect-creation positions were excessively high and repeat defect creation was not feasible, whereas device placement was terminated in one animal because of an excessively high device position accompanied by mitral regurgitation. The VSDs in these dogs were all created via jugular venous interventional puncture, and during the implantation process, the VSRS 3226 occluder (left disc diameter: 32 mm, right disc diameter: 26 mm) was selected for all dogs.

**Figure 3:**
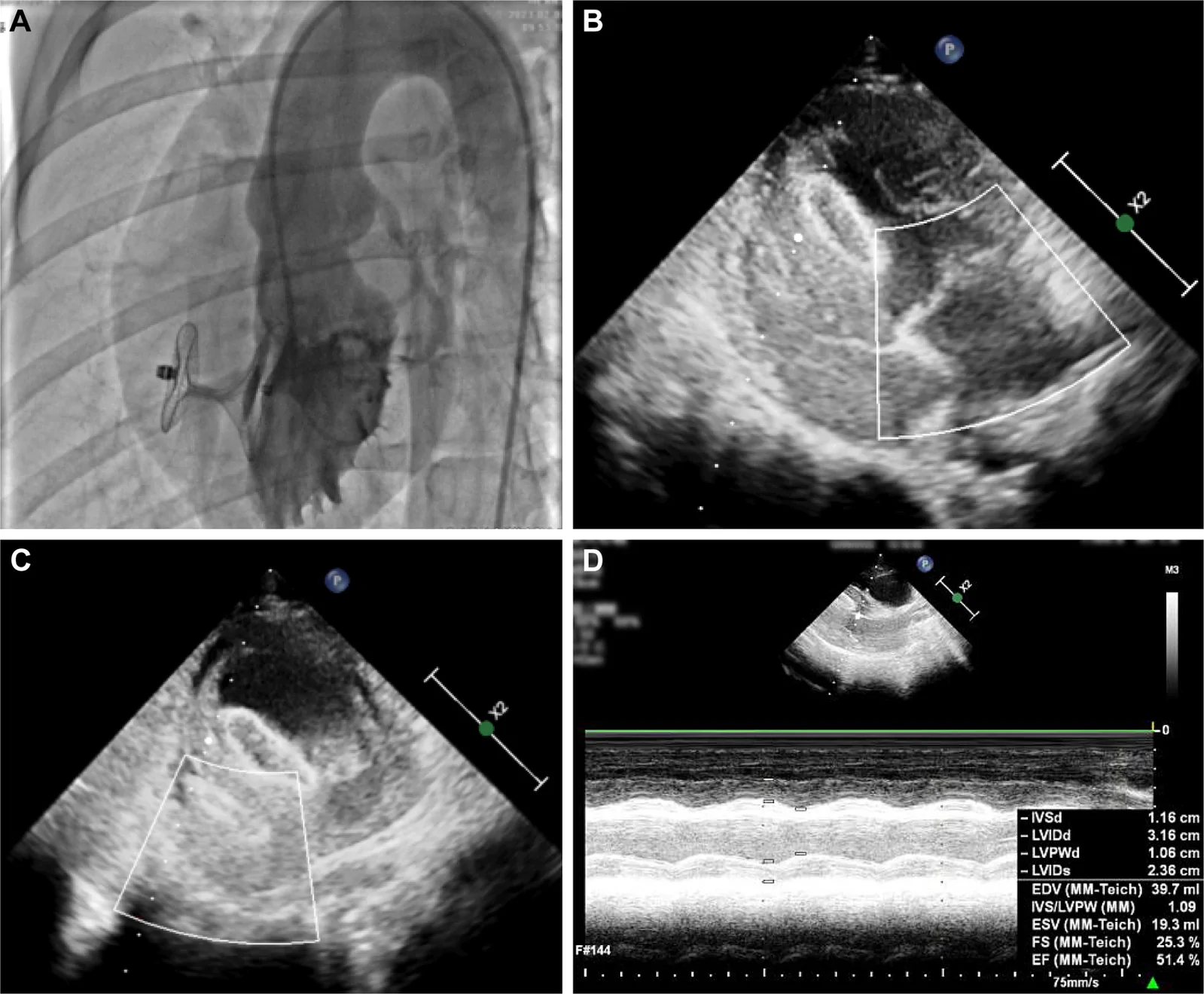
Angiographic and echocardiographic evaluation of experimental animals 60 days after VSRD implantation**. (A)** Follow-up angiography demonstrated no occluder displacement and intact structural integrity of the reconstruction device. **(B)** No tricuspid regurgitation was observed. **(C)** No mitral regurgitation was observed. **(D)** M-mode echocardiographic measurement showed preserved ventricular systolic function, with the measured parameters displayed in the panel.

Successful completion of device placement was therefore achieved in 10 of the 11 animals that underwent a device-placement attempt (90.9%). When all 14 animals that underwent attempted defect creation were used as the denominator, the overall completion rate was 71.4% (10/14). No device structural malfunction or delivery-system failure was documented in the available experimental records. At the end of the experiment, all experimental dogs survived to the predetermined endpoint; no adverse events occurred during the perioperative period or follow-up. No overt abnormalities in mental state or appetite were observed during routine husbandry monitoring; however, no formal neurobehavioral assessment was performed. At the prespecified endpoints, DSA and cardiovascular ultrasound showed stable device position in all animals. Minor residual leakage was qualitatively observed on angiography in four of the five animals in the day-1 group, whereas no leakage was detected in the remaining animal. No residual leakage was detected in any of the five animals in the separate day-60 group. No device migration or structural deformation was observed, and no obvious impairment of mitral valve, tricuspid valve, or cardiac function was detected in this model. Figure 3 demonstrates the follow-up angiography and echocardiographic evaluation conducted 60 days after the implantation of the VSRD. The data pertaining to the animal experiments are shown in Table 1.

**Table 1:** Results of the animal experiments.

Parameter	Result
Animals undergoing attempted VSD-like defect creation	14
Animals included in the final device-performance analysis	10/14 (71.4%)
Male	8 (80)
Weight, kg	31.45 ± 2.57 (28.0-35.0)
Established VSDs	10 (100)
Device Specifications	VSRS 3226
Successful completion of device placement	10/11 (90.9)
Minor residual leakage at day 1	4/5 (80)
Follow-up on 60th day	
Complete closure without residual leakage	5/5 (100)
Adverse events	0 (0)
Thrombosis	0 (0)
Endothelial coverage	Present at 60 d

Values are n, *n* (%), mean ± SD (range), or range. VSDs, ventricular septal defects.

#### 3.1.2 Gross anatomical findings

**Figure 4:**
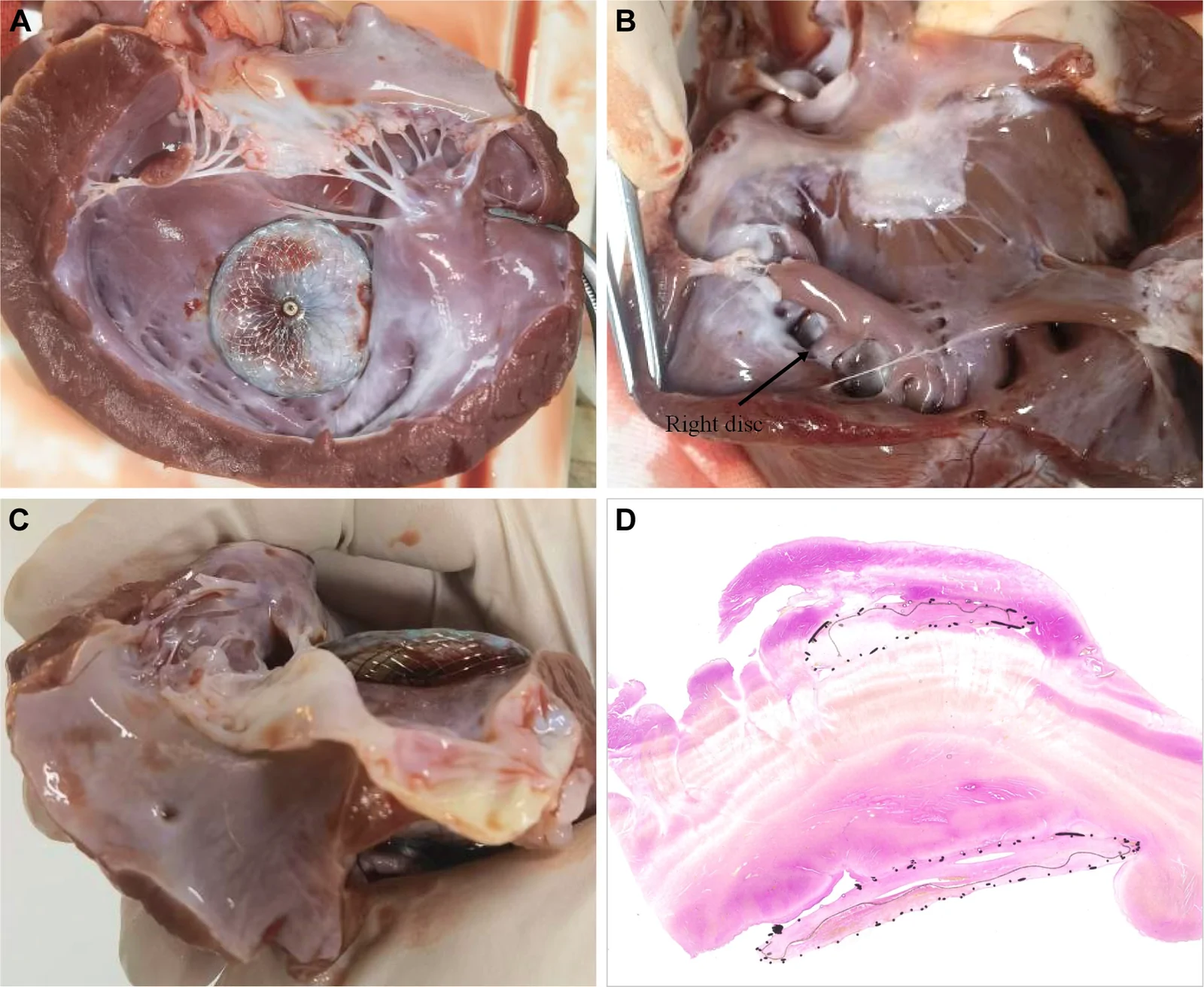
Observations of the occluder and histopathological findings in experimental animals after implantation**. (A)** Left disc of the occluder in the left ventricle. **(B)** Right disc of the occluder in the right ventricle. **(C)** *in vivo* gross observation image after device implantation. **(D)** Representative pathological section of the animal at 60 days.

After implantation of the VSRD, 5 out of 10 experimental dogs were sacrificed for anatomical examination on postoperative day 1, and the remaining 5 were sacrificed on postoperative day 60. Gross anatomical findings revealed that the reconstruction device remained stably positioned with normal morphology at the created ventricular septal defect. The surface of the devices in the 1-day group was clean without attachments, whereas the devices in the 60-day group were covered by tissue consistent with endothelial coverage in this VSD-like model. The disc-shaped structures on both sides of the device were apposed to the surrounding septal tissue, and no vegetations or thrombi were observed. The central portion of the device was measured to be closely apposed, and the overall structure was intact without deformation. Gross examination of the heart and major organs including the liver, spleen, lungs, and kidneys showed no grossly visible infarction lesions or other macroscopic abnormalities, and no tissue damage to the heart caused by device implantation or delivery system manipulation was detected. Figure 4 demonstrates the morphology of the VSRD and results of pathological sections 60 days after implantation.

### 3.2 Short-term results of first-in-human study

**Figure 5:**
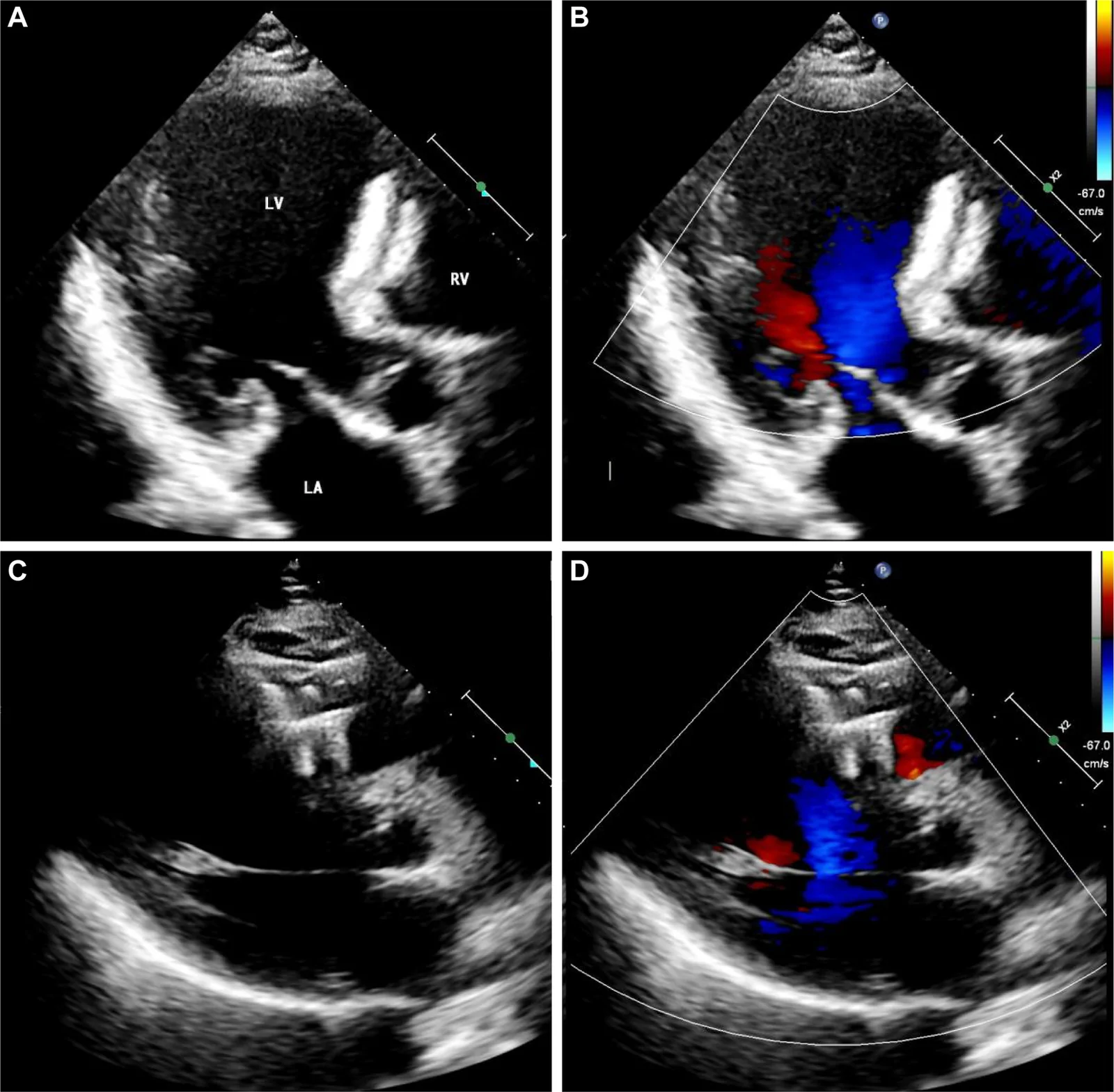
(Panels **A** to **D**) displays the transthoracic echocardiography (TTE) images of a patient at 6 months following implantation of the VSRD: **(A)** represents the apical four-chamber view image acquired via TTE; **(B)** is the color Doppler flow imaging (CDFI) image corresponding to panel A; **(C)** represents the parasternal long-axis view image acquired via TTE; **(D)** is the CDFI image corresponding to panel C. All images indicate that the occluder is accurately positioned and well-apposed, with no significant residual shunt detected.

A total of 4 patients with PIVSR were enrolled during the study period, including 2 men and 2 women, with ages ranging from 59 to 82 years. All four patients had coronary artery disease related to a left anterior descending (LAD) artery culprit lesion and had undergone PCI before VSR closure; no multivessel disease was recorded in the trial dataset. The available coronary treatment records documented successful stent implantation for the culprit LAD lesions, with post-PCI TIMI 3 flow. Among the 4 patients, 1 had concurrent hypertension, 1 had concurrent diabetes mellitus and had experienced cardiogenic shock before surgery, while the remaining patients had no other definite underlying diseases in the trial dataset. Baseline risk features included NYHA class I-II in all patients, Killip class I-II, reduced baseline LVEF in 3 patients, ventricular aneurysm in 3 patients. During the peri-procedural period and follow-up, no patient developed high-grade atrioventricular block, and no patient required permanent pacemaker implantation. No recorded malignant arrhythmia, death, device-related thrombosis, infection, embolization, or vascular access complication was recorded during follow-up. One patient had double defects in the posterior septum, and the other 3 had single defects (1 in the apical posterior septum, 1 in the apical anterior septum, and 1 in the mid-anterior septum), with defect diameters ranging from 7 to 15 mm. The selection of occluders for all patients was performed intraoperatively under the guidance of TTE combined with CT, and each patient underwent VSR closure using only one VSRS series occluder. Occluder models were selected according to defect size using the prespecified sizing rule, while defect location was assessed separately to confirm anatomical suitability and guide procedural planning. Two specifications were used: VSRS 4032 and VSRS 3226. Procedural time ranged from 75 to 200 min. The surgical process was smooth in all four cases, and no severe intraoperative complications occurred. All four procedures met the definition of acute procedural success. All patients had a follow-up duration exceeding 6 months, and no adverse events such as malignant arrhythmia or all-cause mortality were observed during the follow-up period. All patients underwent TTE examinations within 24 h after surgery and at different time points postoperatively; the results showed that the occluders were correctly positioned, residual shunts were all controlled within 2 mm, and no significant device displacement was observed. Available patient-level functional and hemodynamic follow-up information, including hemodynamic support, LVEF trajectory, residual shunt, and 6-month outcomes, is summarized in Table 2. Given the limited sample size, patient-level clinical, anatomical, procedural, and follow-up data are provided in Table 2 rather than interpreted through formal statistical comparison. The surgical and follow-up findings of the patients are shown in Table 2. Figure 5 shows the TTE images of one patient at 6 months after the procedure.

**Table 2:** Patient-Level data in the first-in-human study (*N* = 4).

Parameter	Case 1004	Case 1002	Case 1001	Case 1003
Age/sex	59/F	82/M	67/M	60/F
Admission date	12 Sep 2024	18 Aug 2024	16 May 2024	24 Aug 2024
PCI date, culprit vessel, and post-PCI TIMI flow	14 Sep 2024; LAD; TIMI 3	17 Jul 2024; LAD; TIMI 3	18 May 2024; LAD; TIMI 3	26 Sep 2024; LAD; TIMI 3
Time from AMI to VSR closure	16 d after AMI	38 d after AMI	25 d after AMI	33 d after AMI
Defect anatomy	Single; apical posterior septum	Double; apical posterior septum	Single; apical anterior septum	Single; mid-anterior septum
Defect size	TTE 15 mm; CT 7 mm	TTE 5.8/3.2 mm; CT 9/7 mm	TTE 11 mm; CT 7.8 mm	TTE 15 mm; CT 15 mm
Device size	VSRS 4032	VSRS 3226	VSRS 3226	VSRS 4032
Hemodynamic status	Shock yes; NYHA II; Killip II	Shock no; NYHA I; Killip II	Shock no; NYHA II; Killip II	Shock no; NYHA II; Killip I
IABP/ECMO	No/No	No/No	No/No	No/No
LVEF trajectory	69 -> 67/65/70/NR/65	38 -> 36/40/53/NR/55	32 -> 34/35/38/36.3/40.6	47 -> 40/55/55/55/68
Residual shunt	≤2 mm -> ≤2 mm at 6 mo	≤2 mm -> ≤ 2 mm at 6 mo	≤2 mm -> ≤2 mm at 6 mo	≤2 mm -> ≤2 mm at 6 mo
High-grade AV block	No	No	No	No
Pacemaker implantation	No	No	No	No
Antithrombotic regimen	NR	NR	NR	NR
6-mo outcome	Alive; NYHA I; no malignant arrhythmia/death	Alive; NYHA I; no malignant arrhythmia/death	Alive; NYHA I; no malignant arrhythmia/death	Alive; NYHA I; no malignant arrhythmia/death

Values are presented descriptively. LVEF trajectory is shown as baseline -> 24 h/discharge/30 d/3 mo/6 mo. NR indicates not systematically recorded or not available in the present trial dataset. Exact residual-shunt values beyond the recorded threshold of ≤2 mm and patient-level antithrombotic regimens were not systematically available. AMI, acute myocardial infarction; CT, computed tomography; ECMO, extracorporeal membrane oxygenation; IABP, intra-aortic balloon pump; LAD, left anterior descending artery; LVEF, left ventricular ejection fraction; PCI, percutaneous coronary intervention; TIMI, Thrombolysis in Myocardial Infarction; TTE, transthoracic echocardiography; VSR, ventricular septal rupture; VSRS, Ventricular Septal Reconstruction System.

## 4 Discussion

Among 14 animals undergoing attempted VSD-like defect creation, 10 had anatomically suitable defects and completed device implantation; implantation was also completed in all four selected clinical cases. During the available follow-up, device position remained stable; residual leakage was assessed qualitatively in animals, while residual shunts were ≤2 mm in the clinical cases, supporting preliminary procedural feasibility and short-term performance rather than comparative efficacy or long-term safety.

PIVSR is one of the most life-threatening complications of AMI, characterized by acute onset and rapid progression, which significantly increases the short-term mortality risk of patients. Surgical intervention, while a traditionally effective treatment, is highly invasive, with 10% to 20% of patients experiencing significant residual shunt postoperatively (3). Compared with surgical procedures, interventional therapy offers distinct advantages of minimal invasiveness and rapid postoperative recovery (9, 10). It is particularly suitable for high-risk patients with concurrent cardiac insufficiency and poor surgical tolerance, and has become an important developmental direction in current clinical treatment (2).

However, due to the clinical dilemma of delayed development of PIVSR-specific devices, physicians often use occluders designed for congenital ventricular septal defect (cVSD) for PIVSR treatment. For example, the Amplatzer Atrial Septal Occluder is employed to complete apical PIVSR closure via hybrid techniques (11). Despite achieving certain clinical outcomes, cVSD occluders have inherent limitations in PIVSR treatment, stemming from the fundamental pathological differences between the two conditions: PIVSR is formed by myocardial ischemia, necrosis, and tissue remodeling. It presents as an elliptical and eccentric structure, with dynamic changes in defect size during systole and diastole, extremely irregular edges, and often complex anatomical features such as adjacent independent defects, fenestrated defects combined with locally dilated defects. In contrast, cVSD is mostly regularly circular or elliptical, with a diameter typically ranging only from 3 to 14 mm. Traditional cVSD occluders have fixed geometries optimized for congenital defects; when used for PIVSR, their adaptability may be limited in some irregular or friable anatomies, potentially contributing to residual shunt (12–14).

To address this issue, Abbott has launched the Amplatzer P.I.VSD dedicated device. Tailored to the complex and irregular anatomical characteristics of PIVSR, it optimizes waist length and disc width, with the aim of improving anchoring and occlusion in selected PIVSR anatomies compared with traditional ventricular septal occluders. Clinical data show a technical success rate of 90% and a 1-month postoperative survival rate of 47.6% (8). In that study, intervention timing was heterogeneous, and early closure within 14 days after AMI was observed more frequently among fatal cases than among survivors; the authors also noted that delayed intervention may be influenced by survivorship bias. These observations highlight the importance of intervention timing and baseline clinical status in PIVSR. From a device-design perspective, despite the expanded size range of this series of occluders, certain limitations remain.

Firstly, the relatively fixed structural design results in incomplete adaptability to various defect types. It is difficult to fit extremely irregular, fenestrated, and other complex-shaped defects, potentially leading to poor adherence. Meanwhile, its adaptability to giant defects or special-shaped double defects is still insufficient, making it challenging to fully meet individualized treatment needs. Secondly, the continuous compression exerted by its fixation method on myocardial tissue may affect local blood supply during long-term cardiac systolic and diastolic movements. This is not conducive to myocardial repair and may even increase the risk of long-term complications.

In addition to the Amplatzer P.I.VSD, the custom-made Occlutech PI-VSD occluder has been reported as a purpose-specific, non-CE-marked and non-FDA-approved prototype used on a compassionate-use basis in selected patients with large or complex PIVSR who were unsuitable for standard approved devices or surgery (15–17). Initial case reports and a six-patient series demonstrated technical feasibility; however, device dislodgement or embolization and high mortality among patients with cardiogenic shock indicated the need for further device refinement, careful patient selection, and additional clinical evaluation (15–17). These reports highlight that PIVSR closure remains an unmet clinical need and that further development and evaluation of purpose-specific devices are warranted.

The novel occluder developed in this study was designed to address several of the aforementioned anatomical and mechanical challenges through a dedicated structural design. Briefly, the device uses a narrow-waist, wide-disc configuration with double-disc clamping, which was intended to reduce radial loading at the rupture core and increase the sealing surface around selected defect margins, potentially reducing mechanical stimulation and tension-induced injury to fragile myocardium. The narrow waist precisely fits the core area of the defect, while the wide-edge discs on both sides are designed to increase the sealing surface around selected defect margins, achieving stable fixation through flexible clamping force and avoiding compression injury from traditional rigid fixation. The medical polyester outer membrane isolates direct contact between metal and myocardium, reducing the risk of inflammatory reactions and conduction block. The device specifications cover a wider range, allowing for a 50% expansion relative to the defect diameter, which is consistent with the predefined device size-selection strategy for post-myocardial infarction ventricular septal defects and may help address the size-matching challenges associated with traditional devices (14, 18). This broader coverage may theoretically be advantageous for larger or complex defects; however, this potential application was not evaluated in the present first-in-human study, in which defect diameters were limited to ≤16 mm. Therefore, the narrow waist should not be interpreted as a mechanism that inevitably permits clinically important residual leakage; its function is to minimize radial stress at the rupture core, while the large PET-covered discs provide the major sealing surface. Minor residual jets, when present, were assessed before release and managed by repositioning or recapture/exchange when necessary, followed by serial echocardiographic surveillance if the residual shunt was ≤2 mm. The ≤2-mm threshold was prespecified in the study protocol with reference to criteria used in previous VSD closure studies, in which successful closure was defined as complete closure or a residual shunt ≤2 mm (14).

Results from animal experiments and first-in-human (FIM) observations further support the procedural feasibility and potential clinical value of this device. In the preclinical study, the model should be understood as an interventionally created VSD-like septal defect model. The animal data therefore mainly support deliverability, deployment, anchoring, shunt reduction, device-surface tissue coverage, and short-term local tissue response in a VSD-like model, rather than performance in necrotic, friable, infarcted septal tissue. FIM data showed that all patients treated with this device achieved one-time successful occlusion. All enrolled patients had LAD-related coronary artery disease treated with PCI before closure, and no multivessel disease was documented in the trial dataset. The observed short-term survival and event-free follow-up outcomes are reported descriptively within this selected stabilized cohort and should be confirmed in larger studie s(14)^.^ Notably, in one selected patient with double ventricular septal defects, the wide-edge discs of the occluder covered both defects during a single procedure, suggesting a potential procedural feature in carefully selected anatomically suitable cases. Intraoperative echocardiography and left ventricular angiography are used to assess disc apposition and any minor residual jets before release; when apposition is suboptimal, repositioning, recapture, or device exchange can be performed, and residual shunts ≤2 mm are followed with serial echocardiography. Taken together, the preclinical findings should be interpreted as feasibility and short-term local tissue-response data in a VSD-like model only. Animal health status was assessed through routine husbandry observations rather than standardized behavioral testing; therefore, subtle behavioral changes cannot be excluded.

### 4.1 Study limitations

First, the animal experiments and first-in-human study were designed as exploratory studies with short-term follow-up. The animal study was intended to evaluate device deliverability, deployment, anchoring, shunt reduction, and local tissue response in a controlled VSD-like model, while the first-in-human study was intended to provide initial clinical feasibility observations in selected patients. Therefore, the current data should be interpreted as preliminary feasibility and short-term performance evidence, and longer-term studies are needed to further assess tissue compatibility, device durability, and late clinical outcomes.

Second, the first-in-human study enrolled only 4 selected patients, with relatively limited defect types and follow-up duration. Some patient-level variables, including Qp:Qs, quantitative residual-shunt measurements beyond the recorded threshold of ≤2 mm, and antithrombotic regimens, were not systematically available in the current dataset. Although echocardiographic results relevant to efficacy assessment were evaluated by two independent cardiac imaging physicians according to the protocol, no independent core laboratory was specified, and no data safety monitoring board or clinical event committee was established for this exploratory feasibility study. Larger studies with broader anatomical inclusion and longer follow-up are warranted to evaluate long-term safety, effectiveness, durability, and clinical applicability.

## 5 Conclusions

The VSRD is a novel dedicated reconstruction device designed for post-myocardial infarction ventricular septal rupture (PIVSR). In this small cohort, device implantation was technically successful, and short-term imaging follow-up suggested stable device position and residual shunts no greater than 2 mm; however, these findings should not be interpreted as establishing definitive safety, efficacy, durability, or superiority over existing devices. Larger controlled studies with longer follow-up are needed to evaluate long-term safety, effectiveness, durability, and comparative performance. Taken together, the present preclinical and first-in-human findings support the procedural feasibility of transcatheter closure with this device and provide preliminary short-term observations in this exploratory setting.

## Data Availability

The original contributions presented in the study are included in the article/Supplementary Material, further inquiries can be directed to the corresponding authors.
